# A Direct Comparison of Remote Sensing Approaches for High-Throughput Phenotyping in Plant Breeding

**DOI:** 10.3389/fpls.2016.01131

**Published:** 2016-08-03

**Authors:** Maria Tattaris, Matthew P. Reynolds, Scott C. Chapman

**Affiliations:** ^1^International Maize and Wheat Improvement CenterTexcoco, Mexico; ^2^CSIRO Agriculture, Queensland Bioscience PrecinctQueensland, QLD, Australia

**Keywords:** UAV, multispectral, thermal, indices, airborne imagery, high-throughput phenotyping

## Abstract

Remote sensing (RS) of plant canopies permits non-intrusive, high-throughput monitoring of plant physiological characteristics. This study compared three RS approaches using a low flying UAV (unmanned aerial vehicle), with that of proximal sensing, and satellite-based imagery. Two physiological traits were considered, canopy temperature (CT) and a vegetation index (NDVI), to determine the most viable approaches for large scale crop genetic improvement. The UAV-based platform achieves plot-level resolution while measuring several hundred plots in one mission via high-resolution thermal and multispectral imagery measured at altitudes of 30–100 m. The satellite measures multispectral imagery from an altitude of 770 km. Information was compared with proximal measurements using IR thermometers and an NDVI sensor at a distance of 0.5–1 m above plots. For robust comparisons, CT and NDVI were assessed on panels of elite cultivars under irrigated and drought conditions, in different thermal regimes, and on un-adapted genetic resources under water deficit. Correlations between airborne data and yield/biomass at maturity were generally higher than equivalent proximal correlations. NDVI was derived from high-resolution satellite imagery for only larger sized plots (8.5 × 2.4 m) due to restricted pixel density. Results support use of UAV-based RS techniques for high-throughput phenotyping for both precision and efficiency.

## Introduction

High-throughput phenotyping, particularly through the application of remote sensing tools, offers a rapid and non-destructive approach to plant screening (White et al., 2012). Recent advances in remote sensing technologies as well as in data processing has increased applications in both field and controlled growing conditions (Leinonen and Jones, 2004; Jones et al., 2007; Möller et al., 2007; Swain and Zaman, 2012; Araus and Cairns, 2014) with important consequences for crop improvement.

Remotely sensed spectral readings are based on the interaction between incoming radiation and target objects, resulting in a characteristic signature of reflected light. Such signatures are typically used to calculate spectral indices, which are a function of the light absorption properties of the plant at given wavelengths (e.g., see Tables 7.1–7.3 in Mullan, 2012 and Table 2 in Zarco-Tejada et al., 2013). Two commonly used traits for high-throughput screening are the Normalized Difference Vegetation Index (NDVI), and canopy temperature (CT). NDVI is calculated using wavelengths within the NIR (near infrared) and VIS (visible) regions of the electromagnetic spectrum. NDVI relates to chlorophyll content due to absorption features of the molecule, and hence the photosynthetic capacity of the plant. CT, which is measured from emitted infra-red radiation, can be used as a tool to indirectly evaluate the transpiration rate of a plant (Berliner et al., 1984; Peñuelas et al., 1992). Based mainly on ground based proximal sensing approaches, CT shows a robust association with plant performance, especially under stress, being intimately associated with water status and stomatal conductance (Blum et al., 1982; Berliner et al., 1984; Amani et al., 1996) while NDVI can estimate relative crop biomass at different growth stages (Babar et al., 2006) as well as N deficiency and crop senescence rate (Blum et al., 1982; Reynolds et al., 1994, 1998; Raun et al., 2001; Babar et al., 2006; Olivares-villegas et al., 2007).

Notwithstanding the examples cited above, proximal remote sensing methods can lose precision at high-throughput due to changes in environmental conditions between the start and end of measurements (typically a time period of one to several hours for breeding trials). Satellite imagery has the advantage of covering large areas instantaneously, but generally does not offer the spatial (sub-meter) and temporal (weekly/daily) resolution required for breeding experiments. Low level, airborne remote sensing measurements have the advantage in that resolution is at plot level while at the same time providing the possibility of instantaneously capturing multiple plots at a practical breeding scale at a high temporal resolution (Araus and Cairns, 2014; Chapman et al., 2014).

While a body of literature has shown the value of airborne derived spectral indices to estimate environmentally determined performance traits for a number of crops (Shanahan et al., 2001; Champagne et al., 2002; González-Dugo et al., 2006; Berni et al., 2009; Zhang et al., 2009; Dupin et al., 2011; Swain and Zaman, 2012; Zarco-Tejada et al., 2012), the use of UAVs to increase throughput for breeding purposes and the focus on genetic effects within one agronomic treatment is relatively new (Lelong et al., 2008; Chapman et al., 2014; Díaz-Varela et al., 2015; Zaman-Allah et al., 2015). The UAV approach has obvious potential to increase throughput but the issue of precision relative to other approaches has not been examined. Moreover, data from UAVs have not been compared with satellite derived imagery for phenotyping applications.

The work presented here aims to demonstrate the potential of low level thermal and multispectral UAV imagery and high-resolution multispectral satellite imagery for the derivation of spectral indices of experiments that comprise of 100 s of plots (Table 1) growing in realistic field environments. A methodology was developed with three main objectives. The first was to compare data derived from the UAV with proximal sensors, to determine how well they relate to each other, and their relative ability to predict biomass and yield of wheat. A second objective was to compare data derived from the UAV with satellite imagery for different sized experimental breeding plots, as well as with equivalent data at ground level. The focus of the UAV measurements was the derivation of NDVI and a spectral index relating to canopy temperature, and similarly NDVI and CT were measured using proximal instruments on the ground. For the satellite imagery, NDVI was calculated. A third objective was to evaluate the robustness of the UAV derived indices as selection tools by examining their relationship with crop performance characteristics of different classes of breeding material growing in different simulated target environments. Specifically, the screening traits were measured on advanced breeding lines under optimal, heat stressed, and water deficit conditions, while un-adapted genetic resources were evaluated under water deficit.

**Table 1:** **Details of the five trials under the three environments of DRT (drought), OPT (irrigated) and HOT (hot irrigated)**.

**Trial**	**Germplasm**	**Env**	**Plot size (m)**	**No. of lines/reps**	**Sowing date**	**Harvest date**	**Variable**	**Alternative remote sensing approach**		
								**Proximal date**	**UAV date**	**Satellite date**
Elite OPT	Elite	OPT	8.5 × 2.4	27/3	Nov 2012	May 2013	NDVI	11/3/13	11/3/13	6/4/13
								26/3/13	25/3/13	
Elite HOT 1	Elite	HOT	2.0 × 0.8	30/2	Feb 2012	May 2012	NDVI	15/5/12	11/5/12	
							CT	14/5/12	17/5/12	
Elite HOT 2	Elite	HOT	2.0 × 0.8	60/2	March 2014		NDVI	23/5/14	15/5/14	
								1/6/14	3/6/14	
							CT	13/5/14	13/5/14	
								15/5/14	15/5/14	
								20/5/14	16/5/14	
Elite DRT	Elite	DRT	2.0 × 0.8	50/3	Dec 2012	June 2013	NDVI	14/2/13	13/2/13	
								7/3/13	4/3/13	
							CT	18/2/14	21/2/14	
Gen Res DRT	Un-adapted genetic	DRT	2.0 × 0.8	208/2	Dec 2012	June 2013	NDVI	21/2/13	26/2/13	
	resources						CT	31/3/13	25/3/13	
								7/2/13	22/2/13	

Harvest date indicates the approximate date at which harvest was made for yield and biomass estimates. Measurement dates of ground-based, UAV, and satellite data used for comparisons.

## Materials and methods

### Study site

Trials were located at an experiment station of the International Maize and Wheat Improvement Centre (CIMMYT) in the Sonoran desert, close to Ciudad Obregon, NW Mexico (27°20′ N; 109°54′ W; and 38 m above sea level). Environmental and management details of this area are given in (Sayre et al., 1997). Five trials made up of elite lines and un-adapted genetic resources were studied in three different environments (Table 1); optimal irrigated (OPT), drought stress (DRT) (Gutiérrez-Rodríguez et al., 2004), and hot-irrigated (HOT) (Pinto et al., 2010). The trials named Elite OPT, Elite HOT 1, Elite HOT 2, and Elite DRT are made up of advanced spring wheat lines from CIMMYT adapted to the optimal, hot-irrigated and drought stress environments, respectively. The trial denoted as Gen Res DRT, sown under drought stress, is made up of landraces mainly from Mexico, northern Africa and western central Asia, chosen for potential expression of drought adaptive traits. All trials were sown under an alpha-lattice design, with either two or three replications.

### Proximal data collection

Grain yield (gm^−2^) and dry biomass weight (gm^−2^) were estimated at maturity for each plot following the methods described in Pask et al. (2012) (see Table 1 for harvest dates). Key phenological stages of emergence, heading, anthesis, and physiological maturity were recorded for each plot (Pask et al., 2012).

Canopy temperature (CT) was recorded at ground level using the Sixth Sense LT300 handheld infrared thermometer. Measurements were made along each of the plots from a distance of ~0.5 m above canopy, angled to avoid bare soil (about 60° to nadir) and directed specifically at the part of the plot most exposed to the sun (i.e., with the sun behind observer), when cloud cover was minimal and at times of low wind speed (Pask et al., 2012).

NDVI was measured at ground level with the Trimble Greenseeker 505 Hand-Held active sensor. This instrument emits and measures light at 656 and 774 nm. Measurements were made close to noon, when the plant canopy and soil surface are dry, at about 0.5 m horizontally above the canopy such that the FOV is directly above the plot and centered over the middle row (Pask et al., 2012). NDVI allows for the estimation of vegetation present in each measurement via Equation 1 (Rouse et al., 1973):

(1) $$\begin{array}{l} NDVI=\frac{NIR-R}{NIR+R} \end{array}$$

where *NIR* and *R* are the measured reflectance in the NIR and red spectral bands respectively, (774 and 656 nm for the case of the Greenseeker). Table 1 details the measurement dates for the proximal instruments.

### UAV data collection

**Figure 1:**
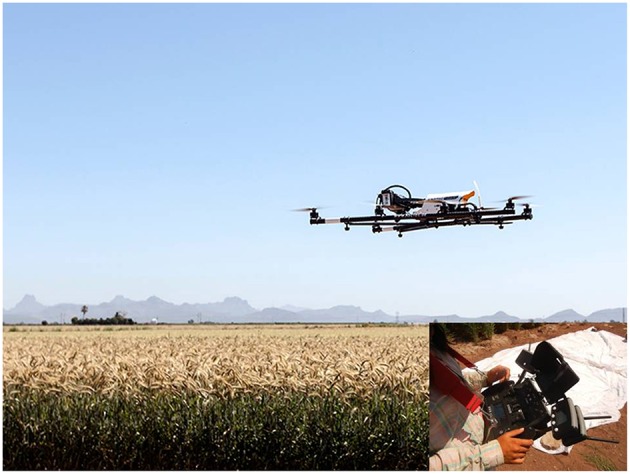
**The airborne remote sensing platform used in this study: The AscTec Falcon 8 Unmanned Aerial Vehicle (UAV), operated with the Mobile Ground Station (inset)**.

**Figure 2:**
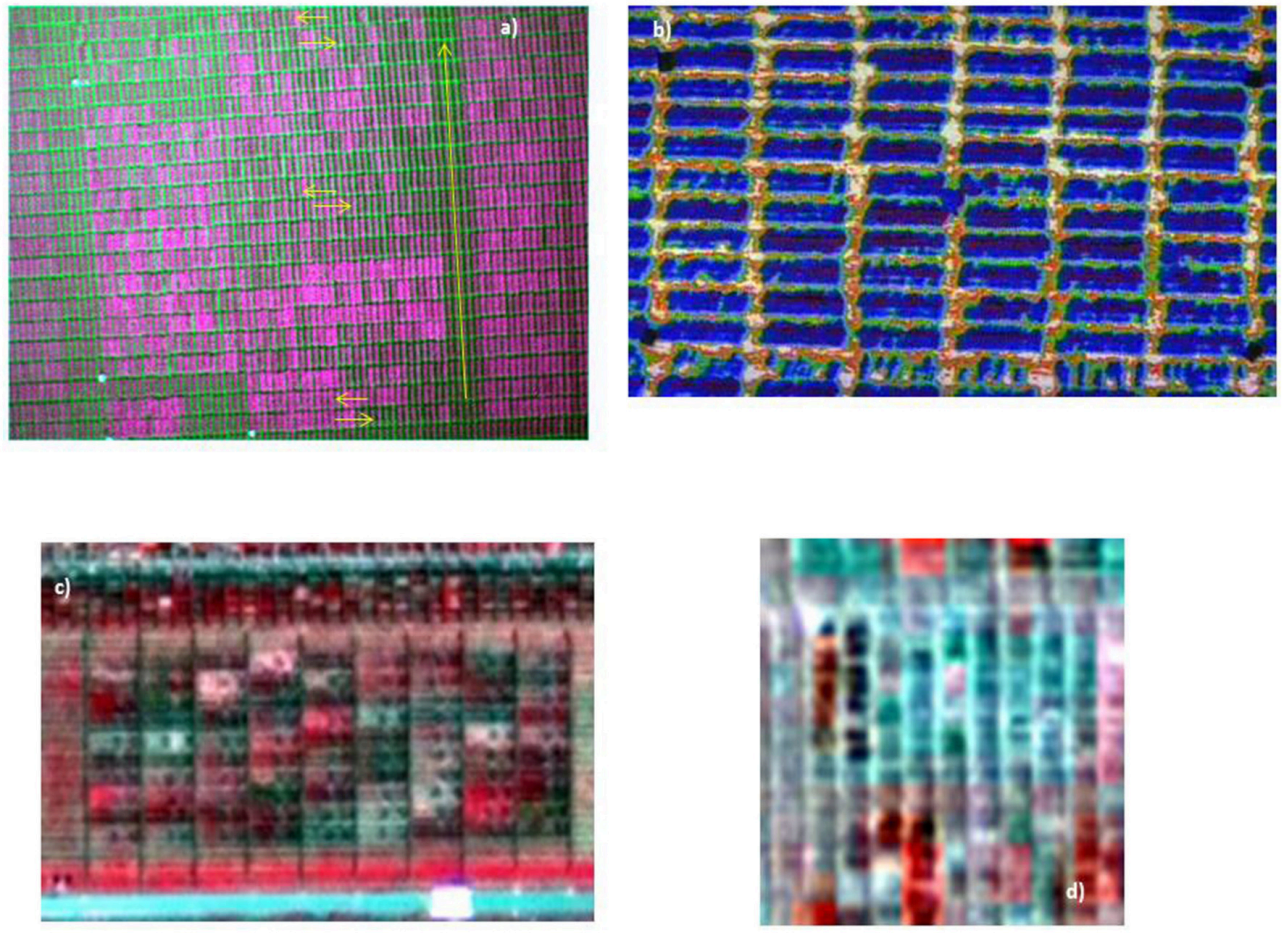
**(A)**. Raw image of Gen Res DRT trial within the drought environment, taken using the ADC Lite Tetracam on the UAV, approximately at 100 m height. Ground dimensions of plots are 2 × 0.8 m, with arrows representing direction of proximal measurements. Assuming a measurement time of 10 s per plot, the time taken to complete measurements using proximal sensors is ~69 min for this trial, compared to several seconds with the UAV. **(B)** Raw image of a “HOT” trial extracted from video footage from the FLIR Tau thermal camera. Flight altitude was ~30 m. Ground dimensions of plots are 2 × 0.8 m. **(C)** Pan-sharpened WV-2 imagery of Elite OPT. Pan-sharpened imagery of a trial containing smaller sized plots in **(D)** did not allow for the extraction of NDVI as plots were mixed within pixels.

Aerial imagery was collected via the AscTec Falcon 8 Unmanned Aerial Vehicle (UAV) (Figure 1). The 8-rotor UAV has a maximum 750 g payload; hence it has the ability to fly small, lightweight instruments. The flight system includes an on-board in-built GPS and a Mobile Ground Station (Figure 1, inset). Aerial images were collected with two cameras mounted separately on the UAV; the Tetracam ADC Lite multispectral camera (2048 × 1536 pixels for Red Green and NIR bands together) and the FLIR Tau 640 LWIR uncooled thermal imaging camera (640 × 512 pixels). See Table 2 for specifications of the cameras. An 8000 mAh lithium battery powers the UAV and cameras, providing ~15-min flight time. Several batteries allow for multiple flights in one session. The ADC Lite Tetracam takes photos in the green, red and NIR regions of the electromagnetic spectrum (Figure 2A), allowing for the calculation of NDVI, while the FLIR thermal camera is used to derive a thermal index relating to the CT of the target plots. In the specification used, the thermal camera records analog video (integrated over 7.5–13 μm), which is subsequently converted to still images for processing (Figure 2B).

**Table 2:** **Specifications of the two cameras mounted on the UAV**.

**Instrument**	**Dims (in)**	**Weight (g)**	**Resolution**	**Spectral range**	**Lens (mm)**	**Pixel size (μm)**
Tetracam ADC lite multispectral camera	4.5 × 3.0 × 2.38	200	2048 × 1536	Green, red, and NIR (TM2, TM3, and TM4)	8.0	3.2
FLIR Tau 640 LWIR uncooled thermal imaging camera	1.74 × 1.75 × 1.18	<72	640 × 512	7.5–13 μm	25	17

### Satellite imagery description

Satellite imagery was obtained from the commercial Digital Globe WorldView-2 (WV-2) satellite, taken on 6th April 2013. The imagery includes an 8-band multispectral image (bands between 396 and 1043 nm) and a panchromatic image (447–808 nm), with a spatial resolution of 0.46 and 1.85 m respectively. The georeferenced, unprocessed images cover ~25 km^2^, including the whole of the CIMMYT research station.

Table 1 presents the measurement dates for the relevant remotely sensed data for each of the trials. Proximal dates were compared based on closest dates available between proximal and airborne data collection.

### Image processing and analysis

#### UAV imagery

Processing was carried out using ENVI version 5.0 (Exelis Visual Information Solutions, Boulder, Colorado). Radiometric distortions, e.g., lens vignetting, were solved by applying a cross track illumination correction, removing any broadband variation without affecting narrowband features. Geometric distortions are corrected using a “warping” procedure, by which an image is resampled to match the geometry of a “base” image or a vector map via the selection of Ground Control Points (GCPs). Images are subsequently mosaicked together by identifying overlapping regions within images.

**Figure 3:**
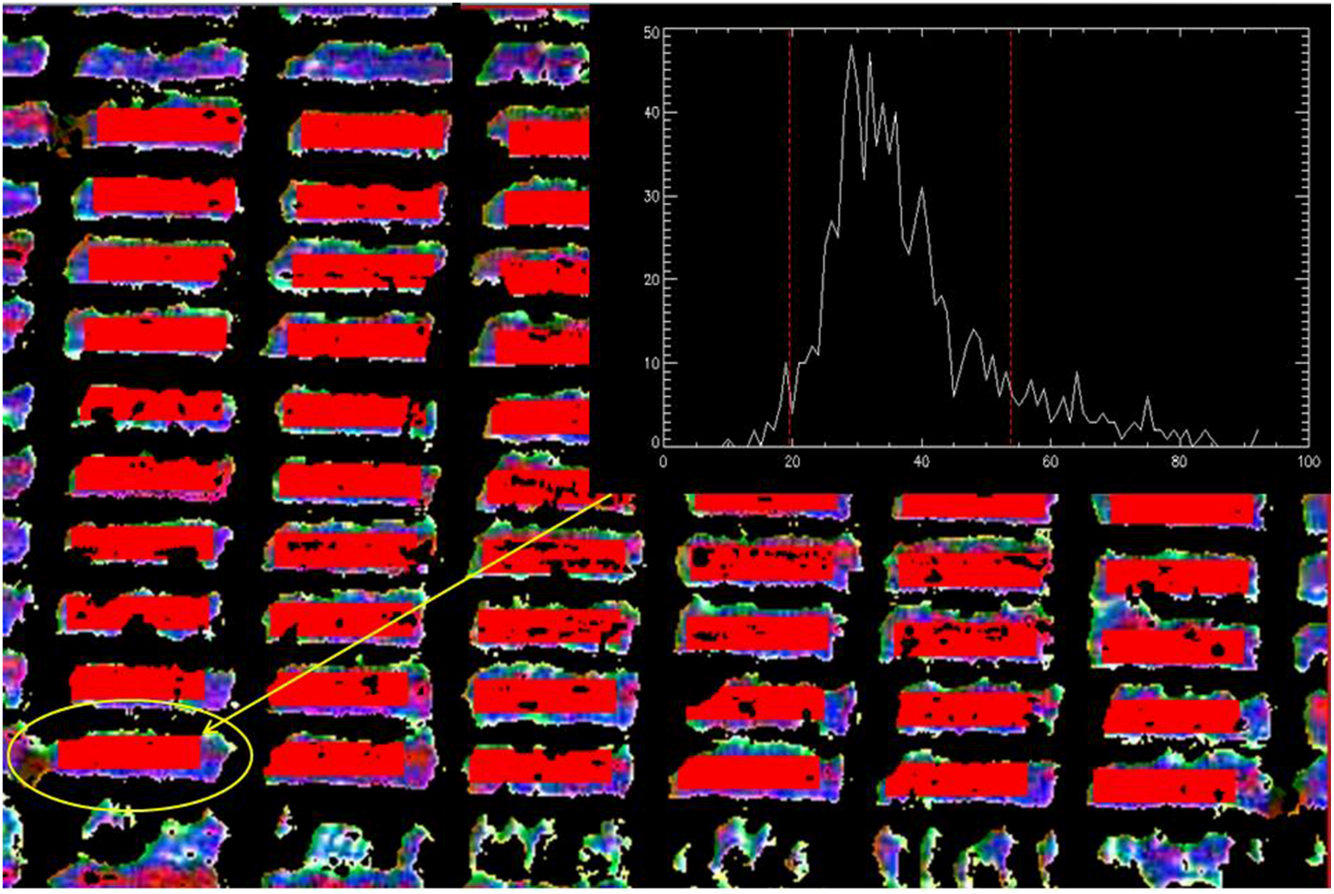
**Example of the image processing using UAV-mounted FLIR Tau image of “HOT” trial shown in Figure 2B where a mask is applied to remove any non-vegetation pixels by applying a threshold for each pixel value**. This is followed by the detection of each plot using pre-defined location parameters (red rectangles) and the removal of high variance pixels (using histogram of the pixel values of each plot). An average of pixel values over each band is taken to get a value per band per plot. This value is then subsequently used to calculate the target indices.

A camera specific mask is applied to the image/mosaic of each trial, via pixel band ratio thresholds, to differentiate between vegetation and non-vegetation pixels, aiming to remove any non-vegetation pixels, such as soil, as different materials can be distinguished by their pixel signal. An algorithm is then applied for the automatic detection of plots using pre-defined parameters by the user, for example the plot size in pixels, and distance between plots in pixels. An average across all bands for each pixel is calculated and pixels within each plot that exhibit high variance are removed, to eliminate any non-vegetation pixels that the mask may have missed, as well as pixel mixing effects. The average of each plot at each band is then taken to derive the target indices at plot level (Figure 3). For the ADC Lite multispectral camera, the NDVI index is calculated as, $\frac{TM4-TM3}{TM4+TM3}$, where *TM4* (≈ 760–900 nm) and *TM3* (≈ 630–690 nm) denote the Landsat bands.

The processed images collected from the thermal camera onboard the UAV were used to derive a temperature index, which relates to the CT of each plot. The temperature index *T*_*I*_ was calculated using the sum of the green and blue bands of the plot averaged values of the processed images acquired from the recorded analog video:

(2) $$\begin{array}{r} T_{\text{1}}=T_{G}+T_{B}\; \end{array}$$

where *T*_*G*_ and *T*_*B*_ are the averaged “plot” values at the green and blue bands respectively.

#### Satellite imagery

The Fast Line-of-sight Atmospheric Analysis of Spectral Hypercubes (FLAASH), an ENVI atmospheric correction tool, was applied to the satellite imagery used here. FLAASH incorporates the Moderate Resolution Atmospheric Transmission V4 (MODTRAN4) radiative transfer model to simulate the spectral radiance at pixel sensor level using user defined input variables. For a detailed description on the methods used by FLAASH see Adler-Golden et al. (1999).

The panchromatic (spatial resolution 0.46 m) and the multispectral imagery (spatial resolution 1.8 m) were fused together to create a single high resolution multispectral image, via the ESRI “pan-sharpening” algorithm, using ESRI ArcMap 10.1 (Mishra and Zhang, 2013). The pan-sharpened image, with a spatial resolution of 0.46 m, was used to derive NDVI (using 833 nm and 659 nm as the NIR and red wavelengths, respectively) as this can then be compared with the NDVI derived from the UAV (ADC Lite), and proximal (GreenSeeker) measurements.

### Statistical analysis

All satellite, airborne and proximal data was spatially corrected for row and column variation across the experiments using Multi Environmental Trial Analysis (META) for SAS (Vargas et al., 2013). Adjusted means were computed individually at each measurement date for the given traits based on the lattice design of the trials. Phenotypic correlations among adjusted genotype means per trial per date were determined to compare the relationship between the airborne, proximal, and agronomic traits. When more than one reading of data was available for both airborne and proximal data, multiple correlations are presented. Statistical analysis was carried out using R 3.1.2 (R Core Team, 2014). Differences between the phenotypic correlations of the proximal and UAV indices against yield or biomass were tested for significance with a Student *t*-test. The Holm-Bonferroni method was applied to the *p*-values of the *t*-tests to account for multiple comparisons (Holm, 1979). In addition, in order to investigate the interactions of the UAV and proximal phenotypic correlations against yield and biomass under the three different environments, for the CT/thermal index and NDVI, a multi-factor analysis of variance (ANOVA) was performed using R.3.1.2.

## Results

### Comparison of data from airborne and proximal sensing approaches

Table 3 shows phenotypic correlations between airborne and proximal sensed data, using mean values of genotypes for both the thermal index and NDVI measured from the UAV compared with the equivalent traits -CT and NDVI- measured using proximal sensors. Correlations between the UAV derived thermal index and the proximal CT are, in general, significant for all trials. This adds confidence to the use of the airborne thermal index. Difficulties can arise when comparing the two different methodologies due to the sensitivity of CT to external environmental factors (time of day, temperature, radiation, wind, irrigation status, VPD, etc.), particularly wind speed. Nonetheless of all the comparisons made, only one did not show a correlation, that between CT and the thermal index for the trial Elite DRT, probably due to variations in wind speed during measurements (as was noted at the time of observation). Significant correlations were also observed between the UAV and proximal NDVI measurements for all trials.

**Table 3:** **Phenotypic correlations between genotype means for the airborne/satellite derived thermal index/NDVI, against the corresponding ground-based CT/NDVI and between the genotypic means for the aerial derived indices and yield/biomass**.

**Trial**	**ENV**	**Variable**	**Alternative remote sensing approach**		**Phenotypic correlation between methodologies**	**Phenotypic correlation with yield**			**Phenotypic correlation with biomass**	
			**Proximal date**	**UAV date**	**Proximal VS. UAV**	**UAV VS. yield**	**Proximal VS. yield**		**UAV VS. biomass**	**Proximal VS. biomass**
Elite OPT	OPT	NDVI	11/3/13	11/3/13	0.78^**^	0.36^+^	0.37^+^		0.45^*^	0.39^*^
			26/3/13	25/3/13	0.79^**^	0.29^+^	0.38^+^		0.52^**^	0.41^*^
Elite HOT 1	HOT	NDVI	15/5/12	11/5/12	0.85^**^	0.75^**^	0.52^**^		0.79^**^	0.58^**^
		CT	14/5/12	17/5/12	0.78^**^	−0.73^**^	−0.56^**^		−0.78^**^	−0.6^**^
Elite HOT 2	HOT	NDVI	23/5/14	15/5/14	0.86^**^	0.51^**^	0.54^**^		0.59^**^	0.65^**^
			1/6/14	3/6/14	0.87^**^	0.64^**^	0.64^**^		0.74^**^	0.73^**^
		CT	13/5/14	13/5/14	0.36^**^	−0.45^**^	−0.34^**^		−0.56^**^	−0.37^**^
			15/5/14	15/5/14	0.4^**^	−0.57^**^	−0.43^**^		−0.57^**^	−0.49^**^
			20/5/14	16/5/14	0.75^**^	−0.62^**^	−0.48^**^		−0.62^**^	−0.55^**^
Elite DRT	DRT	NDVI	14/2/13	13/2/13	0.41^**^	0.56^**^	0.1		–	–
			7/3/13	4/3/13	0.80^**^	0.42^**^	0.27^+^		–	–
		CT	18/2/14	21/2/14	−0.04	−0.41^**^	−0.24^+^		–	–
Gen Res DRT	DRT	NDVI	21/2/13	26/2/13	0.72^**^	0.16^**^	0.25^**^		0.62^**^	0.46^**^
			31/3/13	25/3/13	0.91^**^	0.21^**^	0.14^*^		0.72^**^	0.69^**^
		CT	7/2/13	22/2/13	0.57^**^	−0.44^**^	−0.25^**^		0.1	0.18^+^
**SATELLITE**										
Trial	ENV	Variable	Proximal date	UAV date	Satellite date	SAT VS. proximal		SAT VS. UAV	SAT VS. yield	SAT VS. biomass
Elite OPT	OPT	NDVI	26/3/13	25/3/13	6/4/13	0.85^**^		0.84^**^	0.53^**^	0.58^**^

Also shown are equivalent correlations with ground-based indices.

+, *, ** represent significant levels of 0.1, 0.05, and 0.01 respectively.

### Association of traits with yield and biomass comparing airborne and proximal sensing approaches

Phenotypic correlations were estimated between UAV derived NDVI and the thermal index with both biomass and yield of genotypes (Table 3). For comparison, the corresponding correlation between proximal NDVI and CT with yield and biomass is also shown. Correlations between the UAV derived thermal index and yield/biomass are significant for almost all trials, and are generally larger than the corresponding proximal CT correlations with the yield and biomass. Note that negative correlations were observed between CT/UAV thermal index and yield/biomass as cooler canopies are generally associated with better adaptation. Similarly, the UAV derived NDVI index generally shows stronger correlations with biomass and yield compared with the respective proximal NDVI.

The “Gen Res DRT” trial is made up of diverse genetic resources expressing non-homogeneous height and which are not necessarily well adapted to the photoperiod and other conditions of the screening environment. This could help explain the relatively lower, although still significant, correlations between NDVI and yield for this trial compared to the hot irrigated and drought data sets from elite material, i.e., there was large variation in development stage and morphology (Table 3). The lower correlation between CT and biomass for this trial compared to yield could also be attributed to confounding effects due to variations in height within the trial and their attendant influence on boundary air layers that affect transpiration rate when there is no breeze. Note that this is not the case for NDVI, which is free from such confounding effects.

For the Elite OPT trial, correlations with yield and NDVI are of lower significance (*p* < 0.1) compared to those of the hot irrigated trials, also made up of elite lines (Table 3). However, these results are consistent with previous observations that these techniques are most effective as a selection tools under abiotic stress (Pinto et al., 2010).

When considered together, correlations between the UAV derived indices and yield/biomass were significantly different to the equivalent proximal sensed correlations (*t*-test, *P* = 0.01). When separated into groups, there was significant difference between the UAV and proximal derived correlations with yield and biomass for the following groups CT/thermal index (*t*-test, *P* = 0.01), yield (*t*-test, *P* = 0.05), biomass (*t*-test, *P* = 0.05), and NDVI (*t*-test, *P* = 0.1).

The phenotypic correlations were separated into the three environments (OPT, DRT, and HOT) and a multi-factor ANOVA was performed. For the OPT environment, a significant interaction (*P* = 0.06) was observed between the proximal and UAV phenotypic correlations between yield and biomass, probably associated with the greater biomass correlations, particularly those of the UAV (see Table 3). For the HOT trials, there was a significant difference (*P* = 0.08) between the proximal and UAV phenotypic correlations between yield and biomass for both NDVI and the thermal index/CT. This can be attributed to the higher correlations for the UAV observations. No interaction was observed for the DRT environment, this can be partly explained by un-adapted material in the genetic resource trial, as explained above.

### Satellite imagery

Figure 2C shows the pan-sharpened WV-2 extracted image of Elite OPT. It can be seen that the satellite image provides sufficient resolution for multiple (~20) pixels for each plot and hence the NDVI index was able to be calculated.

Table 3 compares the NDVI calculated from the three methods: space-borne collected WV-2 imagery, low level airborne collected imagery via the UAV and proximal measurements. The proximal and UAV measurements were chosen to be as close as possible to the satellite imagery collection date. It can be seen that the NDVI derived from all methods are well correlated with each other. The correlation between the NDVI from the satellite image and the NDVI from the other two methods gives confidence to the calculation of NDVI from high resolution satellite imagery for plots of the size of those in Elite OPT trial (8.5 × 2.4 m). Also compared in Table 3 is the relationship between each of the NDVI indices and the dry biomass weight and yield measured at maturity for Elite OPT. The NDVI derived from the satellite provide the best correlation with biomass and yield, and proximal NDVI the lowest.

An attempt was made to retrieve NDVI from trials of smaller sized plots. Figure 2D shows an extract of the pan-sharpened WV-2 imagery from an OPT trial with plot size at 2 m × 0.8 m. The resolution of the image prevented the separation of plots due to pixel mixing; hence it was not possible to distinguish between plots.

## Discussion

The results of the current study demonstrate the advantage of airborne remote sensing as a tool to estimate a range of physiological and agronomic traits on a large scale in experimental plots. Proximal measurements have already been proven to predict yield and biomass in wheat (Reynolds et al., 1994, 1998; Aparicio et al., 2000) and are beginning to be used routinely in breeding (Pask et al., 2014). The generally strong correlations presented here between airborne indices and equivalent ground-based CT and NDVI, as well as significant correlations between the airborne indices and yield/biomass, that were generally greater than the equivalent correlations with ground-based measurements, suggest that increased precision results from the use of the indices derived from imagery, particularly in the stressed environments. This is a promising result given the impacts of changing climate and its implications for food security.

Most published work that attempts to thoroughly validate multispectral indices is based on proximal measurements. Errors may be introduced when moving from proximal to aerial measurements at a spatially larger scale, for example atmospheric scattering may cause absorption features of light by pigments to alter, as well as affects related to canopy architecture (angle and area of leaves), water vapor in the atmosphere, background noise and measurement geometry (Suarez et al., 2008; Garbulsky et al., 2011). However, the results presented here demonstrate the potential of low level UAV measurements to indirectly measure yield and biomass in field conditions. The relative precision of airborne measurements can be associated with two main factors. The first is related to reduced errors linked to the ability to remove non-vegetation pixels and other statistical outliers during image analysis (Figure 3). The second is through limiting confounding effects caused by environmental drift, such as changes in temperature, sun angle etc., typically associated with the time taken to make ground based measurements on large trials (Figure 2).

Given that the operation of UAVs is less labor intensive than proximal readings, as well as being free from restrictions associated with access to plots (due to irrigation or application of pesticides, for example), the approach lends itself well to routine measurements including for growth analysis, to measure the evolution of stress, and the application of regression e.g., (Lopes and Reynolds, 2012) or spline (Hurtado et al., 2011) models over time from which additional parameters can be derived to compare treatments and genotypes.

Despite the promising results presented here for NDVI derived from the satellite measurements, it is probably not the most effective tool for this application. While satellite imagery has the advantage of covering vast areas, resolution restricts its application to measurements in which target objects are of a larger scale than the small plots typical of genotypic screening. Furthermore, it is difficult to obtain satellite imagery at frequent time intervals and the option to adjust timing of measurement to avoid cloud cover or other inclement weather conditions is absent.

The fact that the estimates of CT and NDVI were generally better associated with performance traits when measured by UAVs compared to proximal data, under both heat and drought stressed conditions, and in advanced lines as well as unimproved genetic backgrounds, confirms the value of the UAV approach in breeding for climate change, where a new generation of breeding lines must be developed based on extensive screening of plant genetic resources.

## Author contributions

MT collected data, carried out the data analysis, drafted the article and carried out revisions. MR created the experimental design, made suggestions for the data analysis, helped with the draft and carried out critical revisions. SC made suggestions for the data analysis, helped with the draft and carried out critical revisions. All authors gave final approval for publication.

### Conflict of interest statement

The authors declare that the research was conducted in the absence of any commercial or financial relationships that could be construed as a potential conflict of interest.

## References

[B1] Adler-Golden S. M. Matthew M. W. Bernstein L. S. Levine R. Y. Berk A. Richtsmeier S. C. (1999). Atmospheric correction for shortwave spectral imagery based on MODTRAN4. Proc. SPIE 3753 Imaging Spectrometry V. 3753, 61–69. 10.1117/12.366315

[B2] Amani I. Fischer A. Reynolds M. P. (1996). Canopy temperature dpression association with yield of irrigated wheat cultivars in a hot climate. J. Agron. Crop Sci. 176, 119–129. 10.1111/j.1439-037X.1996.tb00454.x

[B3] Aparicio N. Villegas D. Casadesus J. Araus J. L. Royo C. (2000). Spectral vegetation indices as nondestructive tools for determining durum wheat yield. Agron. J. 92, 83–91. 10.2134/agronj2000.92183x

[B4] Araus J. L. Cairns J. E. (2014). Field high-throughput phenotyping: the new crop breeding frontier. Trends Plant Sci. 19, 52–61. 10.1016/j.tplants.2013.09.008 24139902

[B5] Babar M. A. Reynolds M. P. Van Ginkel M. Klatt A. R. Raun W. R. Stone M. L. (2006). Spectral reflectance to estimate genetic variation for in-season biomass, leaf chlorophyll, and canopy temperature in wheat. Crop Sci. 46, 1046–1057. 10.2135/cropsci2005.0211

[B6] Berliner P. Oosterhuis D. M. Green G. C. (1984). Evaluation of the infrared thermometer as a crop stress detector. Agric. For. Meteorol. 31, 219–230. 10.1016/0168-1923(84)90036-4

[B7] Berni J. A. J. Zarco-tejada P. P. J. Suarez L. Fereres E. Member S. Suárez L. (2009). Thermal and narrowband multispectral remote sensing for vegetation monitoring from an unmanned aerial vehicle. IEEE Trans. Geosci. Remote Sens. 47, 722–738. 10.1109/TGRS.2008.2010457

[B8] Blum A. Mayer J. Gozlan G. (1982). Infrared thermal sensing of plant canopies as a screening technique for dehydration avoidance in wheat. Field Crops Res. 5, 137–146. 10.1016/0378-4290(82)90014-4

[B9] Champagne C. M. Staenz K. Bannari A. White H. P. Deguise J. McNairn H. (2002). Estimation of plant water content of agricultural canopies using hyperspectral remote sensing, in 1st International Symposium on Recent Advances in Quantitative Remote Sensing (Valencia).

[B10] Chapman S. C. Merz T. Chan A. Jackway P. Hrabar S. Dreccer M. F. (2014). Pheno-copter: a low-altitude, autonomous remote-sensing robotic helicopter for high-throughput field-based phenotyping. Agronomy 4, 279–301. 10.3390/agronomy4020279

[B11] Díaz-Varela R. de la Rosa R. León L. Zarco-Tejada P. (2015). High-resolution airborne UAV imagery to assess olive tree crown parameters using 3D photo reconstruction: application in breeding trials. Remote Sens. 7, 4213–4232. 10.3390/rs70404213

[B12] Dupin S. Gobrecht A. Tisseyre B. (2011). Airborne thermography of vines canopy: effect of the atmosphere and mixed pixels on observed canopy temperature, in 8 ème Conférence Européenne sur l'Agriculture de Précision (Prague), 1–9.

[B13] Garbulsky M. F. Peñuelas J. Gamon J. Inoue Y. Filella I. (2011). The photochemical reflectance index (PRI) and the remote sensing of leaf, canopy and ecosystem radiation use efficiencies. A review and meta-analysis. Remote Sens. Environ. 115, 281–297. 10.1016/j.rse.2010.08.023

[B14] González-Dugo M. P. Moran M. S. Mateos L. Bryant R. (2006). Canopy temperature variability as an indicator of crop water stress severity. Irrigation Sci. 24, 233–240. 10.1007/s00271-005-0022-8

[B15] Gutiérrez-Rodríguez M. Reynolds M. P. Escalante-Estrada J. A. Rodríguez-González M. T. (2004). Association between canopy reflectance indices and yield and physiological traits in bread wheat under drought and well-irrigated conditions. Aust. J. Agric. Res. 55, 1139–1147. 10.1071/AR04214

[B16] Holm S. (1979). A simple sequentially rejective multiple test procedure. Scand. J. Stat. 6, 65–70.

[B17] Hurtado P. X. Schnabel S. K. Zaban A. Vetelainen M. Virtanen E. Eilers P. H. C. (2011). Dynamics of senescence-related related QTLs in potato. Euphytica 183, 289–302. 10.1007/s10681-011-0464-4

[B18] Jones C. L. Weckler P. R. Maness N. O. Jayasekara R. Stone M. L. Chrz D. (2007). Remote censing to estimate chlorophyll concentration in spinach using multi-spectral plant reflectance. Am. Soc. Agric. Biol. Eng. 50, 2267–2273. 10.13031/2013.24079

[B19] Leinonen I. Jones H. G. (2004). Combining thermal and visible imagery for estimating canopy temperature and identifying plant stress. J. Exp. Bot. 55, 1423–1431. 10.1093/jxb/erh146 15133055

[B20] Lelong C. C. D. Burger P. Jubelin G. Roux B. Labbé S. Baret F. (2008). Assessment of unmanned aerial vehicles imagery for quantitative monitoring of wheat crop in small plots. Sensors 8, 3557–3585. 10.3390/s8053557 PMC3675559 27879893

[B21] Lopes M. S. Reynolds M. P. (2012). Stay-green in spring wheat can be determined by spectral reflectance measurements (normalized difference vegetation index) independently from phenology. J. Exp. Bot. 63, 3789–3798. 10.1093/jxb/ers071 22412185 PMC3388823

[B22] Mishra R. K. Zhang Y. (2013). A comparison of commercial Pan-sharpening techniques for HR Satellite imagery, in 2013 Esri International User Conference (Esri UC) (San Diego, CA).

[B23] Möller M. Alchanatis V. Cohen Y. Meron M. Tsipris J. Naor A.. (2007). Use of thermal and visible imagery for estimating crop water status of irrigated grapevine. J. Exp. Bot. 58, 827–838. 10.1093/jxb/erl115 16968884

[B24] Mullan D. (2012). Spectral radiometry, in Physiological Breeding I: Interdisciplinary Approaches to Improve Crop Adaptation, eds Reynolds M. P. Pask A. Mullan D. (CIMMYT), 69–80.

[B25] Olivares-villegas J. J. Reynolds M. P. McDonald G. K. (2007). Drought-adaptive attributes in the Seri/Babax hexaploid wheat population. Funct. Plant Biol. 34, 189–203. 10.1071/fP06148 32689345

[B26] Pask A. Joshi A. K. Manes Y. Sharma I. Chatrath R. Singh G. P. (2014). A wheat phenotyping network to incorporate physiological traits for climate change in South Asia. Field Crops Res. 168, 156–167. 10.1016/j.fcr.2014.07.004

[B27] Pask A. Pietragalla J. Mullan D. (2012). Physiological Breeding II: A Field Guide to Wheat Phenotyping. Mexico: CIMMYT.

[B28] Peñuelas J. Savé R. Marfa O. Serrano L. (1992). Remotely measured canopy temperature of greenhouse strawberries as indicator of water status and yield under mild and very mild water stress condictions. Agric. For. Meteorol. 58, 63–77.

[B29] Pinto R. S. Reynolds M. P. Mathews K. L. McIntyre C. L. Olivares-Villegas J. J. Chapman S. C. (2010). Heat and drought adaptive QTL in a wheat population designed to minimize confounding agronomic effects. Theor. Appl. Genet. 121, 1001–1021. 10.1007/s00122-010-1351-4 20523964 PMC2938441

[B30] Raun W. R. Solie J. B. Johnson G. V. Stone M. L. Lukina E. V. Thomason W. E. (2001). In-season prediction of potential grain yield in winter wheat using canopy reflectance. Agron. J. 93, 131 10.2134/agronj2001.931131x

[B31] R Core Team (2014). R: A language and environment for statistical computing. Vienna: R Foundation for Statistical Computing Available online at: http://www.R-project.org/.

[B32] Reynolds M. P. Balota M. Delgado M. I. B. Amani I. Fischer R. A. (1994). Physiological and morphological traits associated with spring wheat yield under hot, irrigated conditions. Aust. J. Plant Physiol. 21, 717–730. 10.1071/PP9940717

[B33] Reynolds M. P. Singh R. P. Ibrahim A. Ageeb O. A. A. Larque-Saavedra A. Quick J. S. (1998). Evaluating physiological traits to complement empirical selection for wheat. Euphytica 100, 85–94. 10.1023/A:1018355906553

[B34] Rouse J. Haas R. Schell J. Deering D. (1973). Monitoring vegetation system in the great plains with ERTs, in Third ERTS Symposium (Washington, DC), 309–317.

[B35] Sayre K. Rajaram S. Fischer R. A. (1997). Yield potential progress in short bread wheats in Northwest Mexico. Crop Sci. 37, 36–42. 10.2135/cropsci1997.0011183X003700010006x

[B36] Shanahan J. F. Schepers J. S. Francis D. D. Varvel G. E. Wilhelm W. (2001). Use of remote-sensing imagery to estimate corn grain yield. Agronomy 93, 583–589. 10.2134/agronj2001.933583x

[B37] Suarez L. Zarco-Tejada P. J. Sepulcre-Cantó G. Perez-Priego O. Miller J. R. Jiménez-Muñoz J. C. (2008). Assesing canopy PRI for water stress detection with diurnal airborne imagery. Remote Sens. Environ. 112, 560–575. 10.1016/j.rse.2007.05.009

[B38] Swain K. C. Zaman Q. U. (2012). Rice “Crop monitoring with unmanned helicopter remote sensing images,” in Remote Sensing of Biomass - Principles and Applications, ed Fatoyinbo T. (Rijeka: InTech), 254–272.

[B39] Vargas M. Combs E. Alvarado G. Atlin G. Mathews K. Crossa J. (2013). Meta: a suite of sas programs to analyze multienvironment breeding trials. Agron. J. 105, 11–19. 10.2134/agronj2012.0016

[B40] White J. W. Andrade-Sanchez P. Gore M. A. Bronson K. F. Coffelt T. A. Conley M. M. (2012). Field-based phenomics for plant genetics research. Field Crops Res. 133, 101–112. 10.1016/j.fcr.2012.04.003

[B41] Zaman-Allah M. Vergara O. Araus J. L. Tarekegne A. Magorokosho C. Zarco-Tejada P. J.. (2015). Unmanned aerial platform-based multi-spectral imaging for field phenotyping of maize. Plant Methods 11, 35. 10.1186/s13007-015-0078-2 26106438 PMC4477614

[B42] Zarco-Tejada P. J. González-Dugo V. Berni J. A. J. (2012). Fluorescence, temperature and narrow-band indices acquired from a UAV platform for water stress detection using a micro-hyperspectral imager and a thermal camera. Remote Sens. Environ. 117, 322–337. 10.1016/j.rse.2011.10.007

[B43] Zarco-Tejada P. J. Morales A. Testi L. Villalobos F. J. (2013). Spatio-temporal patterns of chlorophyll fluorescence and physiological and structural indices acquired from hyperspectral imagery as compared with carbon fluxes measured with eddy covariance. Remote Sens. Environ. 133, 102–115. 10.1016/j.rse.2013.02.003

[B44] Zhang H. Lan Y. Lacey R. E. Hoffmann W. C. Huang Y. (2009). Analysis of vegetation indices derived from aerial multispectral and ground hyperspectral data. Int. J. Agric. Biol. Eng. 2, 33–40. 10.3965/j.issn.1934-6344.2009.03.033-040

